# Machine learning-based nomogram: integrating MRI radiomics and clinical indicators for prognostic assessment in acute ischemic stroke

**DOI:** 10.3389/fneur.2024.1379031

**Published:** 2024-06-12

**Authors:** Kun Guo, Bo Zhu, Rong Li, Jing Xi, Qi Wang, KongBo Chen, Yuan Shao, Jiaqi Liu, Weili Cao, Zhiqin Liu, Zhengli Di, Naibing Gu

**Affiliations:** ^1^Xi'an Central Hospital, Xi’an, China; ^2^China-Japan Union Hospital of Jilin University, Changchun, China; ^3^Tongchuan Mining Bureau Central Hospital, Tongchuan, China

**Keywords:** acute ischemic stroke, radiomics, multi-parametric MRI, prognosis, nomogram, machine learning

## Abstract

**Background:**

Acute Ischemic Stroke (AIS) remains a leading cause of mortality and disability worldwide. Rapid and precise prognostication of AIS is crucial for optimizing treatment strategies and improving patient outcomes. This study explores the integration of machine learning-derived radiomics signatures from multi-parametric MRI with clinical factors to forecast AIS prognosis.

**Objective:**

To develop and validate a nomogram that combines a multi-MRI radiomics signature with clinical factors for predicting the prognosis of AIS.

**Methods:**

This retrospective study involved 506 AIS patients from two centers, divided into training (n = 277) and validation (*n* = 229) cohorts. 4,682 radiomic features were extracted from T1-weighted, T2-weighted, and diffusion-weighted imaging. Logistic regression analysis identified significant clinical risk factors, which, alongside radiomics features, were used to construct a predictive clinical-radiomics nomogram. The model’s predictive accuracy was evaluated using calibration and ROC curves, focusing on distinguishing between favorable (mRS ≤ 2) and unfavorable (mRS > 2) outcomes.

**Results:**

Key findings highlight coronary heart disease, platelet-to-lymphocyte ratio, uric acid, glucose levels, homocysteine, and radiomics features as independent predictors of AIS outcomes. The clinical-radiomics model achieved a ROC-AUC of 0.940 (95% CI: 0.912–0.969) in the training set and 0.854 (95% CI: 0.781–0.926) in the validation set, underscoring its predictive reliability and clinical utility.

**Conclusion:**

The study underscores the efficacy of the clinical-radiomics model in forecasting AIS prognosis, showcasing the pivotal role of artificial intelligence in fostering personalized treatment plans and enhancing patient care. This innovative approach promises to revolutionize AIS management, offering a significant leap toward more individualized and effective healthcare solutions.

## Highlights

- High predictive accuracy for AIS prognosis.
- Integrates MRI radiomics with clinical factors.
- Utilizes advanced machine learning techniques.
- Provides a validated clinical-radiomics nomogram.
- Facilitates personalized AIS management.

## 1 Introduction

Ischemic stroke remains a formidable public health concern due to its high incidence, mortality, and morbidity rates, exerting a profound impact on society, families, and the affected individuals (1). Despite concerted efforts in recent years toward the management, treatment, and prevention of ischemic stroke, a significant proportion of patients fail to receive timely and effective intervention. This failure is often attributed to delayed recognition of symptoms, a lack of awareness regarding the urgency of medical care, and the unavailability of adequate facilities in primary healthcare settings, leading to varying extents of neurological deficits. The cornerstone of acute ischemic stroke treatment in the acute phase includes intravenous alteplase thrombolysis (2, 3) and mechanical thrombectomy (4). However, the application of intravenous thrombolysis is constrained by a narrow therapeutic time window, and stringent inclusion and exclusion criteria limit mechanical thrombectomy. Recent observations suggest a shift toward an increasing incidence of stroke among younger populations, a trend linked to improved living standards and heightened work-related stress (5, 6). Factors such as the timing of intervention, location and volume of the infarct, and post-stroke treatment and rehabilitation efforts are pivotal in determining patient outcomes and survival rates (7). Consequently, the accurate prediction of acute ischemic stroke prognosis becomes essential for evaluating the severity, identifying potential adverse outcomes, gauging rehabilitation prospects, and enhancing doctor-patient communication and clinical decision-making processes.

When cerebrovascular diseases are suspected or need exclusion, Computed Tomography Angiograms (CTA) and Magnetic Resonance Angiograms (MRA) have demonstrated high specificity and sensitivity. However, Digital Subtraction Angiograms (DSA) remains the definitive gold standard, providing unparalleled diagnostic insight. Despite its utility in detailing intravascular conditions, DSA’s invasiveness and radiation exposure constrain its widespread clinical application.

Developing non-invasive methodologies with minimal radiation exposure is imperative in clinical practice to mitigate these limitations. Such advancements aim to improve the evaluation of treatment outcomes and prognostic accuracy in acute cerebral infarction.

Predictive models that amalgamate clinical observations, imaging findings, laboratory data, and other variables are instrumental in predictive assessment. These models enable comprehensive evaluations of rehabilitation prospects, survival rates, and disease incidence through mathematical and statistical approaches. A pioneering effort in this domain was conducted by Karen C. Johnston’s team in 2000 (8), utilizing the NIH Stroke Scale (NIHSS), Barthel Index (BI), and Glasgow Coma Scale (GCS) to gauge acute ischemic stroke prognosis with promising results. Furthering this initiative, they integrated NIHSS scores and CT infarct volumes to adeptly predict patient outcomes at 3 months. Zhao et al. (9) further explored 30-day survival prediction in acute ischemic stroke patients by analyzing post-stroke blood routine and biochemical markers, including Neutrophil-to-Lymphocyte Ratio (NLR), Prognostic Nutritional Index (PNI), Systemic Immune-Inflammation Index (SII), and Risk Assessment (RA).

Radiomics, employing sophisticated image processing to extract detailed features from imaging studies of acute ischemic stroke patients, unveils in-depth insights into pathophysiological alterations. This technique enhances early diagnosis, disease type determination, precise lesion localization and quantification, and fosters accurate prognosis evaluation and treatment outcome assessment (10–13).

Clinical radiomics models, leveraging features derived from diffusion-weighted imaging (DWI), fluid-attenuated inversion recovery (FLAIR), and apparent diffusion coefficient (ADC) scans, have shown commendable efficacy in prognosticating outcomes for patients with acute ischemic stroke (14, 15). Despite these advancements, the utilization of imaging attributes and clinical data in appraising treatment effectiveness and forecasting the prognosis of acute ischemic stroke remains underexploited. Notably, a blend of T1-weighted images (T1w), T2-weighted images (T2w), and DWI is prevalently employed for assessing patients post-onset. This fact highlights the critical need for an exhaustive amalgamation of various imaging techniques to refine the precision and utility of predictive models in determining acute ischemic stroke outcomes. The potential to enhance predictive accuracy and clinical decision-making through such integrated models is vast yet underleveraged.

The objective of this research is to develop a model that effectively combines T1-weighted (T1w), T2-weighted (T2w), and diffusion-weighted imaging (DWI) features with pertinent clinical parameters. This model explores the associations between imaging characteristics and crucial clinical information, enhancing our understanding of acute ischemic stroke. The primary goal is to create an accurate and individualized decision support system that enriches the treatment process for patients experiencing acute ischemic stroke. We anticipate facilitating significantly improved patient outcomes by achieving this integration, ultimately benefiting those impacted by this condition.

## 2 Materials and methods

### 2.1 Subjects

This retrospective study recruited participants from two healthcare institutions, Xi’an Central Hospital (Center 1) and Tongchuan Mining Bureau Central Hospital (Center 2), with ethical approval from the respective hospitals’ Ethics Committees by the Declaration of Helsinki. A collective cohort of 506 patients diagnosed with Acute Ischemic Stroke (AIS) was retrospectively analyzed across both centers during the period from January to December 2021. Eligibility criteria for inclusion comprised admission within 24 h following symptom onset, an initial assessment using the National Institutes of Health Stroke Scale (NIHSS) upon admission, and undergoing diffusion-weighted imaging (DWI) within the first 72 h post-symptom onset.

**Figure 1:**
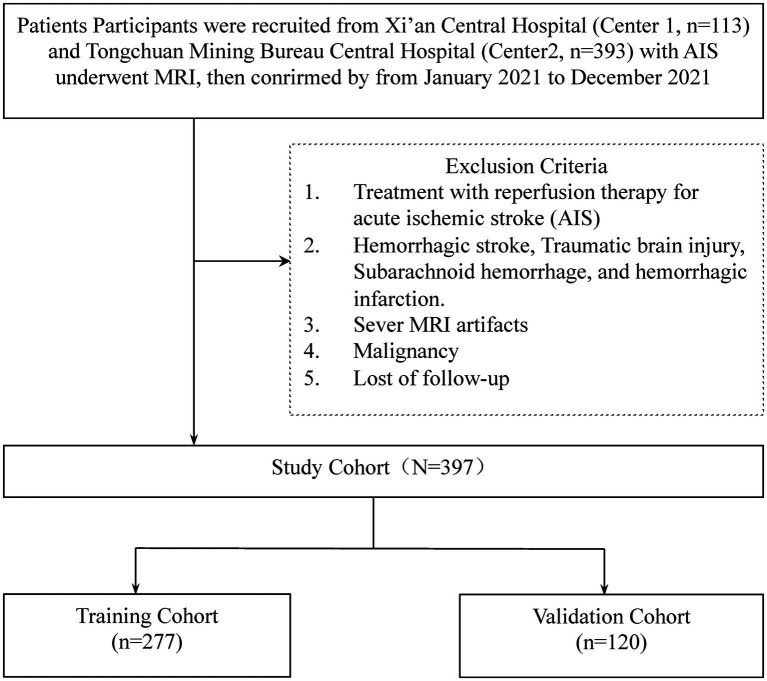
The workflow of the patient selection.

Exclusion criteria were defined to omit patients who underwent reperfusion therapies for AIS, including intravenous thrombolysis with recombinant tissue plasminogen activator (rt-PA), urokinase (UK), and tenecteplase (TNK-tPA), as well as those who received bridging therapy (mechanical thrombectomy) or endovascular treatments (Center 1: *n* = 17, Center 2: *n* = 31). Given the unavailability of UK and TNK-tPA at the study sites, these treatments were not considered. Further exclusions applied to patients with hemorrhagic stroke, traumatic brain injury, subarachnoid hemorrhage, and hemorrhagic infarction (Center 1: *n* = 1, Center 2: *n* = 2), those presenting severe MRI artifacts (Center 1: *n* = 1, Center 2: *n* = 5), diagnosed with malignancies (Center 1: *n* = 0, Center 2: *n* = 1), or lost to follow-up (Center 1: *n* = 10, Center 2: *n* = 41; Figure 1).

Baseline clinical data, encompassing demographics, medical history (e.g., hypertension, hyperlipidemia, hyperuricemia, hypoproteinemia, hyperhomocysteinemia, diabetes, smoking, drinking, prior stroke, atrial fibrillation, coronary artery disease, chronic heart failure, arthrosis, hemadostenosis), TOAST classification, along with an extensive set of laboratory parameters and imaging features, were meticulously extracted from medical records. Two experienced neurologists, each with a decade of practice and blind to clinical and imaging data, conducted structured telephone interviews to determine patients’ modified Rankin Scale (mRS) scores 6 months after hospital discharge, categorizing prognosis into favorable (mRS ≤ 2) and adverse (mRS > 2) outcomes. Although pre-stroke mRS scores are insightful for assessing baseline functionality, their exclusion aims to assure data integrity and minimize bias. The study’s emphasis on uniformly assessed, quantifiable factors across all participants enhances the predictive model’s validity, striving to eliminate confounding influences and bolster the reliability of the findings.

### 2.2 Image data acquisition

MRI scans were performed on all participants within 72 h post-symptom onset using the EXCITE HD 1.5 T MRI system by GE Healthcare, Milwaukee, WI, United States. Transverse T1-weighted fast spin echo (FSE) imaging was executed with specific parameters: TR/TE = 2,259/25.4 ms, slice thickness/gap = 5/1.5 mm, bandwidth = 244 Hz/Px, FOV = 240 × 240 mm^2^, and acceleration factor (R) = 2. For T2-weighted FSE imaging, the settings were TR/TE = 5,582/111 ms, slice thickness/gap = 5/1.5 mm, bandwidth = 244 Hz/Px, FOV = 240 × 240 mm^2^, and R = 3. Diffusion-weighted imaging (DWI) utilized single-shot echo planar imaging (SS-EPI) with TR/TE = 3,203/83.9 ms, slice thickness/gap = 5/1.5 mm, bandwidth = 3,906 Hz/Px, FOV = 240 × 240 mm^2^, R = 2, and b-values of 0 and 1,000 s/mm^2^ (Table 1).

**Table 1:** Comparison of MRI sequence parameters.

MRI sequence	T1w	T2w	DWI
TR/TE(ms)	2,259/25.4	5,582/111	3,203/83.9
Slice Thickness/Gap(mm)	5/1.5	5/1.5	5/1.5
Bandwidth(Hz/Px)	244	244	3,906
FOV(mm)	240*240	240*240	240*240
Acceleration Factor(R)	2	3	2
b-values(s/mm)	/	/	0, 1,000

### 2.3 VOI delineated and radiomics feature extraction

**Figure 2:**
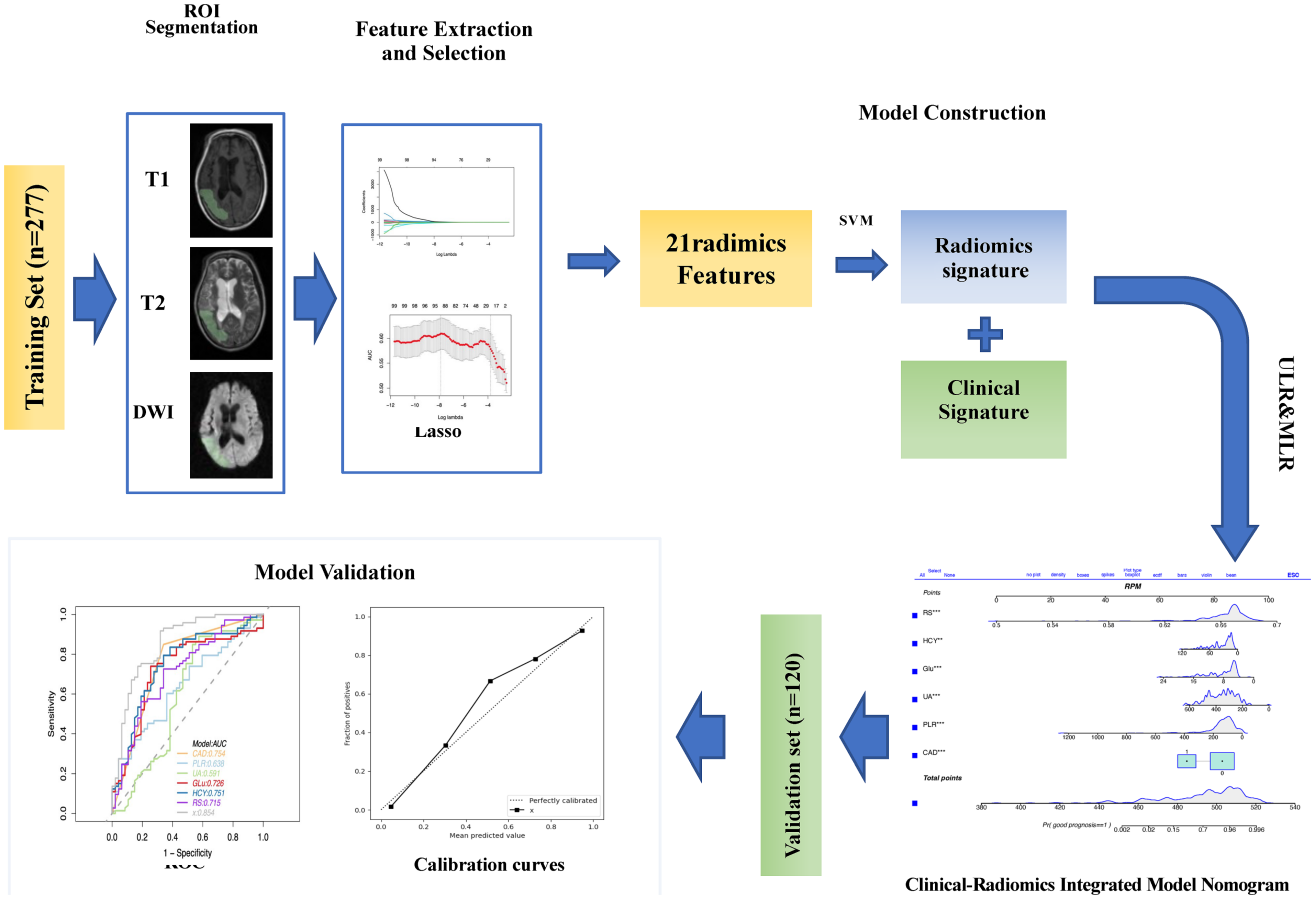
The workflow of the study is as follows: ROI (Region of Interest) segmentation was performed using 3D Slicer. The images were preprocessed for feature extraction. After feature evaluation and model construction, clinical radiomic signatures and imaging radiomic signatures were generated and used to construct a clinical-radiomic model. The performance of this model in predicting the prognosis of acute ischemic stroke (AIS) was validated in the validation set.

Volumes of Interest (VOIs) were meticulously delineated using 3D-Slicer Software, while Pyradiomics software (version 3.0.1) facilitated the computation of radiomics features, adhering to the Image Biomarker Standardization Initiative’s guidelines. A radiologist, blind to the patient’s clinical information, performed the initial segmentation of MRI images. These segmentations were then reviewed and refined by a senior neuroradiologist with extensive experience. The segmentation process targeted the entire infarct region. Utilizing PyRadiomics, various radiomics features were extracted from these VOIs, including shape-based, first-order statistical, and several gray-level matrix features, from T1-weighted, T2-weighted, and diffusion-weighted images, totaling 4,682 features. This methodical extraction process ensures a comprehensive analysis of imaging data, which is crucial for evaluating acute ischemic stroke prognosis (Figure 2).

### 2.4 Features selection

Given the high-dimensional nature of radiomics features in acute ischemic stroke (AIS) analysis, the study aimed to pinpoint features significantly correlated with outcome predictions in the training cohort. Initial feature selection was performed using a U-test, setting a *p*-value threshold 0.05 to filter out non-significant and redundant features. Further refinement involved a correlation analysis to eliminate features with a correlation coefficient above 0.9. The Least Absolute Shrinkage and Selection Operator (LASSO) algorithm was then utilized to finalize feature selection, identifying the most predictive features through fivefold cross-validation.

### 2.5 Prediction development and diagnostic validation

Univariable logistic regression identified potential clinical predictors of AIS outcomes within the training cohort. Subsequently, significant predictors were analyzed through multivariable logistic regression, employing backward stepdown selection to isolate independent clinical predictors. These findings were presented as odds ratios with corresponding 95% confidence intervals, forming the basis of a multivariable clinical prediction model. The model combined these independent clinical predictors with the radiomics signature to create a comprehensive clinical-radiomics model, which underwent rigorous evaluation through ROC curve analysis and other statistical measures to assess its discriminative performance.

### 2.6 Clinical usefulness and calibration curves of the clinical-radiomics model

The clinical-radiomics model’s calibration was examined using calibration curves alongside the Hosmer-Lemeshow test to determine the model’s fit accuracy.

### 2.7 Statistics analysis

The study used statistical methods to analyze demographic and clinical data, including independent *t*-tests for normally distributed data, Mann–Whitney U tests for non-normally distributed data, and chi-square tests for categorical variables. The predictive performance of clinical, radiomics, and clinical-radiomics models was evaluated through Receiver Operating Characteristic (ROC) curves. Model comparisons were conducted using the Delong test. Calibration of the clinical-radionics model was assessed with calibration curves and the Hosmer-Lemeshow test. Statistical analyses were conducted in R software, with significance determined by a two-tailed *p*-value of <0.05.

## 3 Results

### 3.1 Baseline characteristics of patients

The baseline characteristics of patients within both the training and validation groups are detailed in Table 2. Analysis revealed no statistically significant disparities between the cohorts (*p* > 0.05), indicating comparable baseline conditions. Within the training cohort, patients experiencing unfavorable outcomes (modified Rankin Scale, mRS, > 2) accounted for 66.78% (182 out of 277), while in the validation group, this proportion stood at 60.83% (73 out of 120).

**Table 2:** The characteristic of 21 final feature for radiomics assessment.

Radiomic features
T1_original_shape_SurfaceArea
T1_original_firstorder_Energy
T1_wavelet.LLH_ngtdm_Coarseness
T1_wavelet.LHL_glcm_Idmn
T1_wavelet.LHH_glcm_Idmn
T1_wavelet.HLH_glcm_ClusterShade
T1_wavelet.HHL_glrlm_LongRunEmphasis
T1_exponential_glszm_ZoneEntropy
T1_exponential_gldm_DependenceEntropy
T1_squareroot_glcm_ClusterShade
T1_gradient_glrlm_LongRunEmphasis
T2_original_ngtdm_Strength
T2_wavelet.LLL_firstorder_90Percentile
T2_wavelet.LLL_firstorder_InterquartileRange
T2_square_ngtdm_Strength
DWI_log.sigma.1.0.mm.3D_glcm_DifferenceVariance
DWI_wavelet.LHL_firstorder_Mean
DWI_wavelet.LHL_firstorder_Median
DWI_wavelet.LHL_glcm_InverseVariance
DWI_wavelet.HLH_firstorder_Skewness
DWI_wavelet.HHH_firstorder_Kurtosis

### 3.2 Radiomics feature selection and LASSO logistic regression findings

**Figure 3:**
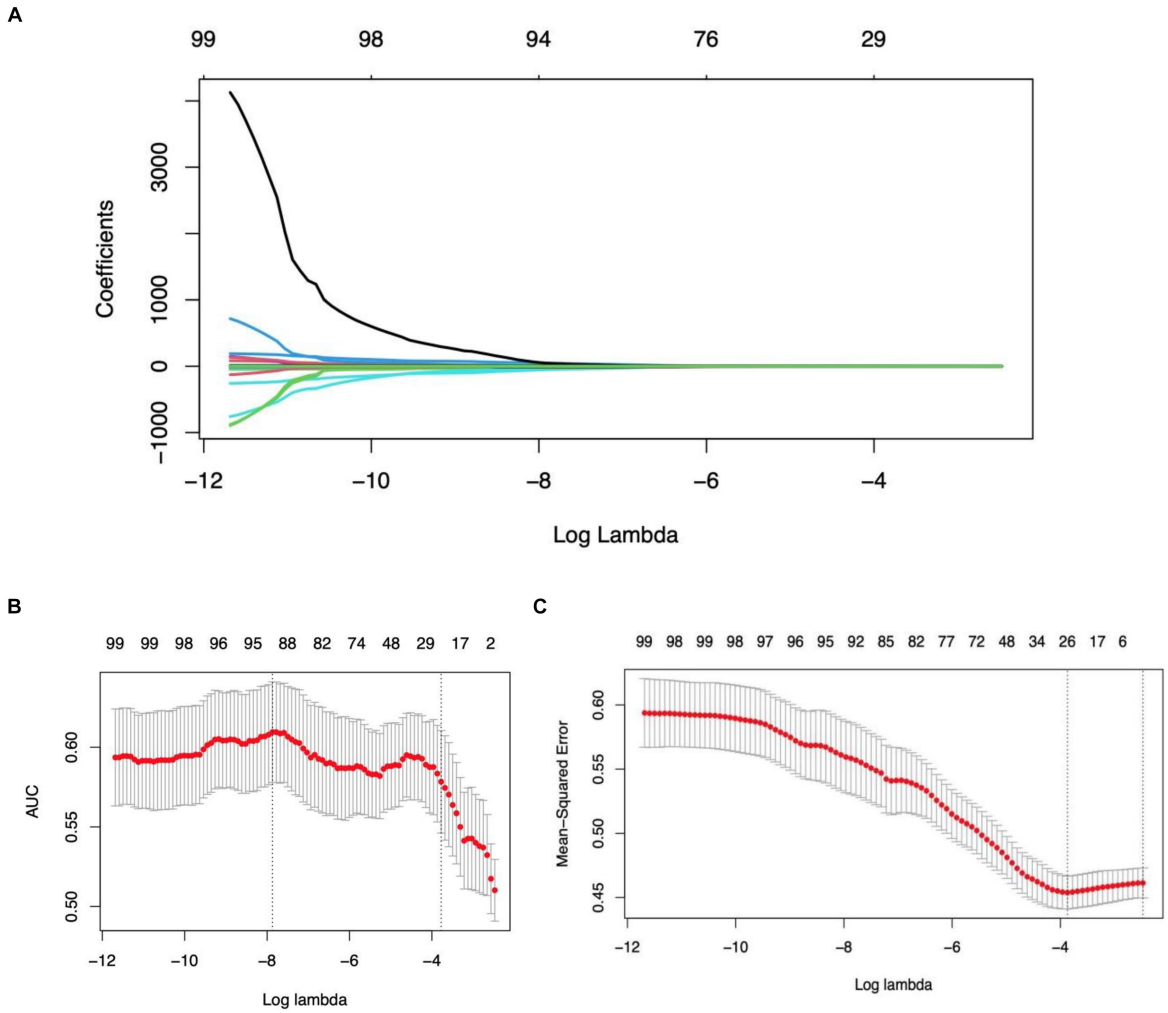
Illustration of the LASSO model’s application, employing a tuning parameter (lambda) and utilizing five-fold cross-validation based on both the minimum criteria and one standard error (1se), facilitating the selection of radiomics features during the feature selection phase.

In refining our dataset, redundant features were eliminated through a U-test and Spearman correlation analysis, narrowing down the selection from an initial pool of 4,682 image features extracted from the Volumes of Interest (VOIs) to 791 radiomic features. This step was essential for enhancing the dataset’s manageability and relevance. Following this initial reduction, the Least Absolute Shrinkage and Selection Operator (LASSO) logistic regression method was applied to further distill these features, focusing on identifying those of optimal predictive value. Determining the most appropriate lambda value was crucial in this context; to this end, five-fold cross-validation was utilized, selecting a lambda value within one standard error of the minimum. Through meticulous selection, a final set of 21 radiomic features was identified (as detailed in Table 3). This rigorous methodology underscores the accuracy and efficacy of the resulting predictive model (illustrated in Figure 3).

**Table 3:** Baseline characteristics of patients in the training and validation cohorts.

	Training cohort			Validation cohort		
	(*n* = 277)			(*n* = 120)		
	Poor	Good	p.overall	Poor	Good	p.overall
	(mRS > 2)	(mRS ≤ 2)		(mRS > 2)	(mRS ≤ 2)	
	*n* = 95	*n* = 182		*n* = 47	*n* = 73	
Age	68.0 [58.5;77.5]	68.0 [60.0;75.0]	0.477	71.0 [62.5;81.0]	69.0 [64.0;78.0]	0.647
Gender:			0.824			0.146
Female	18 (18.9%)	38 (20.9%)		19 (40.4%)	19 (26.0%)	
Male	77 (81.1%)	144 (79.1%)		28 (59.6%)	54 (74.0%)	
Hypertension:			0.003			0.013
No	10 (10.5%)	49 (26.9%)		3 (6.38%)	19 (26.0%)	
Yes	85 (89.5%)	133 (73.1%)		44 (93.6%)	54 (74.0%)	
Hyperlipemia:			<0.001			0.001
No	14 (14.7%)	121 (66.5%)		15 (31.9%)	48 (65.8%)	
Yes	81 (85.3%)	61 (33.5%)		32 (68.1%)	25 (34.2%)	
Hyperuricemia:			<0.001			<0.001
No	22 (23.2%)	150 (82.4%)		22 (46.8%)	62 (84.9%)	
Yes	73 (76.8%)	32 (17.6%)		25 (53.2%)	11 (15.1%)	
Hypoproteinemia:			0.052			0.066
No	77 (81.1%)	164 (90.1%)		35 (74.5%)	65 (89.0%)	
Yes	18 (18.9%)	18 (9.89%)		12 (25.5%)	8 (11.0%)	
Hyperhomocysteinemia:			<0.001			0.04
No	7 (7.37%)	65 (35.7%)		10 (21.3%)	30 (41.1%)	
Yes	88 (92.6%)	117 (64.3%)		37 (78.7%)	43 (58.9%)	
Diabetes:			<0.001			<0.001
No	21 (22.1%)	127 (69.8%)		14 (29.8%)	57 (78.1%)	
Yes	74 (77.9%)	55 (30.2%)		33 (70.2%)	16 (21.9%)	
Smoking history:			<0.001			<0.001
No	29 (30.5%)	140 (76.9%)		26 (55.3%)	65 (89.0%)	
Yes	66 (69.5%)	42 (23.1%)		21 (44.7%)	8 (11.0%)	
Drinking history:			<0.001			<0.001
No	42 (44.2%)	166 (91.2%)		27 (57.4%)	71 (97.3%)	
Yes	53 (55.8%)	16 (8.79%)		20 (42.6%)	2 (2.74%)	
Stroke history:			0.12			0.135
No	49 (51.6%)	113 (62.1%)		19 (40.4%)	41 (56.2%)	
Yes	46 (48.4%)	69 (37.9%)		28 (59.6%)	32 (43.8%)	
AF^*^			1			1
No	87 (91.6%)	168 (92.3%)		44 (93.6%)	69 (94.5%)	
Yes	8 (8.42%)	14 (7.69%)		3 (6.38%)	4 (5.48%)	
CAD^*^			<0.001			<0.001
No	30 (31.6%)	146 (80.2%)		16 (34.0%)	62 (84.9%)	
Yes	65 (68.4%)	36 (19.8%)		31 (66.0%)	11 (15.1%)	
AS^*^			<0.001			0.013
No	18 (18.9%)	95 (52.2%)		10 (21.3%)	33 (45.2%)	
Yes	77 (81.1%)	87 (47.8%)		37 (78.7%)	40 (54.8%)	
Hemadostenosis:			<0.001			0.006
No	20 (21.1%)	105 (57.7%)		10 (21.3%)	35 (47.9%)	
Yes	75 (78.9%)	77 (42.3%)		37 (78.7%)	38 (52.1%)	
ADL^*^	55.0 [40.0;60.0]	80.0 [65.0;100]	<0.001	55.0 [42.5;62.5]	80.0 [65.0;100]	<0.001
NHISS^*^	7.00 [5.00;9.50]	2.00 [1.00;3.00]	<0.001	7.00 [5.00;11.5]	2.00 [1.00;3.00]	<0.001
TOAST^*^			0.007			0.636
Large artery atherosclerosis	8 (8.42%)	7 (3.85%)		0 (0.00%)	2 (2.74%)	
Cardioembolism	72 (75.8%)	138 (75.8%)		39 (83.0%)	60 (82.2%)	
Small vessel occlusion	9 (9.47%)	35 (19.2%)		4 (8.51%)	8 (11.0%)	
Other determined etiology	6 (6.32%)	2 (1.10%)		3 (6.38%)	3 (4.11%)	
Undetermined etiology				1 (2.13%)	0 (0.00%)	
KWST^*^			<0.001			0.001
0	1 (1.05%)	4 (2.20%)		0 (0.00%)	2 (2.74%)	
1	59 (62.1%)	162 (89.0%)		33 (70.2%)	67 (91.8%)	
2	24 (25.3%)	11 (6.04%)		6 (12.8%)	3 (4.11%)	
3	7 (7.37%)	3 (1.65%)		5 (10.6%)	0 (0.00%)	
4	2 (2.11%)	1 (0.55%)		3 (6.38%)	1 (1.37%)	
5	2 (2.11%)	1 (0.55%)				
WBC	10.9 [8.39;12.7]	6.78 [5.56;8.34]	<0.001	11.2 [8.09;13.4]	6.63 [5.55;8.49]	<0.001
NEU	5.53 [4.18;7.18]	4.94 [3.62;6.43]	0.116	6.71 [4.72;8.57]	4.99 [3.61;6.62]	0.005
LYM	0.99 [0.89;1.69]	1.71 [1.30;2.19]	<0.001	1.03 [0.87;1.50]	1.57 [1.24;2.18]	<0.001
NLR^*^	4.73 [2.85;6.42]	2.83 [1.90;4.05]	<0.001	6.03 [3.40;9.48]	3.22 [1.97;4.73]	<0.001
MON	0.43 [0.33;0.64]	0.47 [0.35;0.62]	0.104	0.45 [0.35;0.58]	0.44 [0.35;0.56]	0.796
MLR^*^	0.37 [0.24;0.50]	0.28 [0.20;0.36]	<0.001	0.45 [0.30;0.65]	0.28 [0.20;0.39]	0.001
Hb	145 [135;153]	143 [132;154]	0.468	140 [132;149]	145 [133;156]	0.285
HCT	0.44 [0.41;0.50]	0.44 [0.41;3.69]	0.629	0.42 [0.40;0.45]	0.43 [0.40;0.49]	0.541
PLT	179 [148;219]	178 [147;220]	0.772	180 [130;204]	189 [133;219]	0.292
SII^*^	842 [441;1,287]	459 [298;686]	<0.001	858 [534;1,750]	548 [280;895]	0.002
PLR^*^	150 [98.8;212]	103 [71.9;143]	<0.001	138 [102;228]	115 [74.2;168]	0.011
TBIL	17.8 [13.4;24.0]	16.4 [12.9;21.5]	0.257	19.1 [15.9;26.4]	15.7 [12.3;21.4]	0.011
Alb	38.7 [36.5;42.2]	39.3 [37.3;42.5]	0.309	38.9 [36.7;40.9]	38.9 [36.2;41.8]	0.828
Glb	22.0 [20.0;26.8]	22.4 [19.4;27.3]	0.9	22.5 [19.9;26.0]	22.8 [20.2;26.2]	0.94
ALT	17.0 [12.0;23.0]	16.5 [12.0;22.8]	0.515	16.0 [12.0;23.5]	17.0 [10.0;21.0]	0.735
AST	20.0 [17.0;24.5]	18.5 [16.0;23.0]	0.245	19.0 [15.0;26.5]	18.0 [16.0;23.0]	0.577
AST/ALT	1.38 [1.00;2.25]	1.73 [1.10;2.31]	0.072	1.88 [1.09;2.48]	2.00 [1.20;2.62]	0.55
Urea	5.21 [4.46;6.23]	5.26 [4.48;6.22]	0.873	5.68 [4.73;6.61]	5.78 [4.76;6.75]	0.919
Cr	67.0 [56.5;77.0]	66.6 [55.0;77.8]	0.783	64.0 [57.0;74.6]	63.0 [57.0;74.0]	0.906
UA	409 (104)	324 (88.2)	<0.001	362 (115)	327 (85.9)	0.074
TC	4.35 [3.51;5.38]	4.06 [3.50;4.94]	0.15	4.35 (1.11)	4.40 (1.11)	0.823
PNI^*^	44.9 [42.2;49.3]	47.9 [43.4;52.3]	0.016	45.0 [41.9;49.0]	49.0 [41.8;54.2]	0.09
TG	1.97 [1.05;3.12]	1.35 [0.99;1.95]	0.001	1.34 [0.92;2.42]	1.25 [0.93;1.74]	0.154
HDL	0.98 [0.82;1.15]	1.05 [0.91;1.28]	0.005	1.12 [0.92;1.27]	1.09 [0.96;1.31]	0.807
LDL	2.89 [2.18;3.48]	2.31 [1.70;2.92]	<0.001	2.36 [1.79;3.42]	2.44 [1.92;3.18]	0.517
Glu	11.2 [7.25;13.9]	5.68 [5.04;7.74]	<0.001	11.9 [6.66;13.6]	5.86 [5.14;6.98]	<0.001
HCY	45.2 [24.7;56.2]	19.6 [14.2;29.4]	<0.001	43.7 [19.2;54.5]	16.7 [13.1;22.9]	<0.001
K	3.96 (0.38)	4.08 (0.44)	0.018	4.05 (0.40)	4.09 (0.42)	0.564
Na	141 [139;143]	141 [139;143]	0.532	142 [140;143]	141 [139;143]	0.833
Cl	106 [103;108]	106 [104;108]	0.402	106 [104;108]	105 [103;108]	0.739
Ca	2.25 (0.14)	2.25 (0.14)	0.903	2.25 [2.13;2.33]	2.23 [2.15;2.32]	0.899
P	0.98 (0.19)	0.99 (0.21)	0.595	0.98 (0.22)	0.99 (0.22)	0.859
PT	10.8 [10.2;11.4]	10.9 [10.2;11.6]	0.683	11.0 [10.3;11.9]	10.7 [10.2;11.4]	0.359
INR	0.95 [0.89;1.00]	0.95 [0.89;1.03]	0.663	0.97 [0.90;1.08]	0.95 [0.88;0.99]	0.121
APTT	26.0 [24.4;28.9]	26.8 [24.0;30.4]	0.438	25.9 [23.1;30.2]	26.2 [23.9;28.9]	0.821
TT	16.9 [15.2;17.9]	16.6 [15.2;17.9]	0.489	16.6 [15.4;17.9]	16.8 [15.9;17.6]	0.604
FIB	2.85 [2.33;3.38]	2.82 [2.35;3.36]	0.832	2.61 [2.27;3.36]	2.66 [2.22;3.20]	0.526
D-Dimer	0.46 [0.23;1.17]	0.34 [0.21;0.64]	0.04	0.56 [0.29;1.17]	0.40 [0.26;0.69]	0.089
FDP	1.60 [1.00;3.60]	1.36 [0.90;2.30]	0.06	1.50 [1.13;3.20]	1.30 [0.70;2.20]	0.08
EF	57.0 [55.0;65.0]	58.0 [55.0;64.0]	0.996	61.0 [55.5;65.0]	60.0 [55.0;65.0]	0.363
LEVF	110 [101;124]	115 [105;131]	0.078	114 [104;125]	113 [105;127]	0.815
LVDD^*^	30.0 [28.0;32.0]	30.0 [28.0;32.0]	0.66	30.0 [28.0;32.0]	30.0 [28.0;33.0]	0.462
LVSD^*^	44.0 [41.0;46.0]	45.0 [43.0;48.0]	0.012	45.0 [42.0;47.0]	45.0 [43.0;48.0]	0.599
HR	76.0 [69.5;81.5]	76.0 [67.0;82.0]	0.704	77.0 [71.0;80.0]	75.0 [66.0;82.0]	0.344
SBP	150 [138;162]	145 [133;155]	0.03	151 (21.1)	150 (19.8)	0.879
DBP	88.0 [78.0;95.5]	86.0 [78.0;94.8]	0.3	85.0 [78.5;96.0]	86.0 [76.0;93.0]	0.743
Ht	170 [160;175]	170 [165;174]	0.717	170 [160;172]	168 [162;172]	0.901
IdealWt	64.2 [57.0;68.0]	65.0 [59.8;66.5]	0.97	62.0 [56.0;66.1]	63.5 [57.8;65.8]	0.696
Wt	65.0 [60.0;74.0]	66.5 [61.2;74.0]	0.894	65.0 [56.5;70.0]	65.0 [55.0;70.0]	0.859
BMI	23.8 [21.5;25.4]	23.2 [22.0;25.0]	0.251	22.6 [20.7;25.2]	23.0 [20.3;24.8]	0.866
GNRI^*^	62.2 [58.6;67.1]	63.1 [59.8;67.6]	0.288	62.4 [58.8;65.6]	62.4 [57.5;67.3]	0.782
**GNRI grade:**						
1	0 (0.00%)	1 (0.55%)				
3	95 (100%)	181 (99.5%)		47 (100%)	73 (100%)	
RS^*^	0.66 [0.65;0.67]	0.67 [0.66;0.67]	<0.001	0.66 [0.65;0.67]	0.67 [0.66;0.68]	<0.001

AF, atrial fibrillation; CAD, coronary artery disease; AS, atherosclerosis; ADL, activities of daily living; NIHSS, National Institutes of Health Stroke Scale; TOAST, Trial of Org 10172 in Acute Stroke Treatment; KWST, Kubota Water Swallowing Test; NLR, neutrophil-to-lymphocyte ratio; MLR, monocyte-to-lymphocyte ratio; SII, systemic immune-inflammation index; PLR, platelet-to-lymphocyte ratio; PNI, prognostic nutritional index; LVDD, left ventricular end-diastolic diameter; LVSD, left ventricular end-systolic diameter; GNRI, geriatric nutritional risk index; RS, radiomic signature. Data for categorical variables are expressed in terms of the number of patients (percentage), and continuous variables are represented by median [interquartile range]. The *p*-value is used for group comparisons.

### 3.3 Establishment and performance of the clinical features

**Figure 4:**
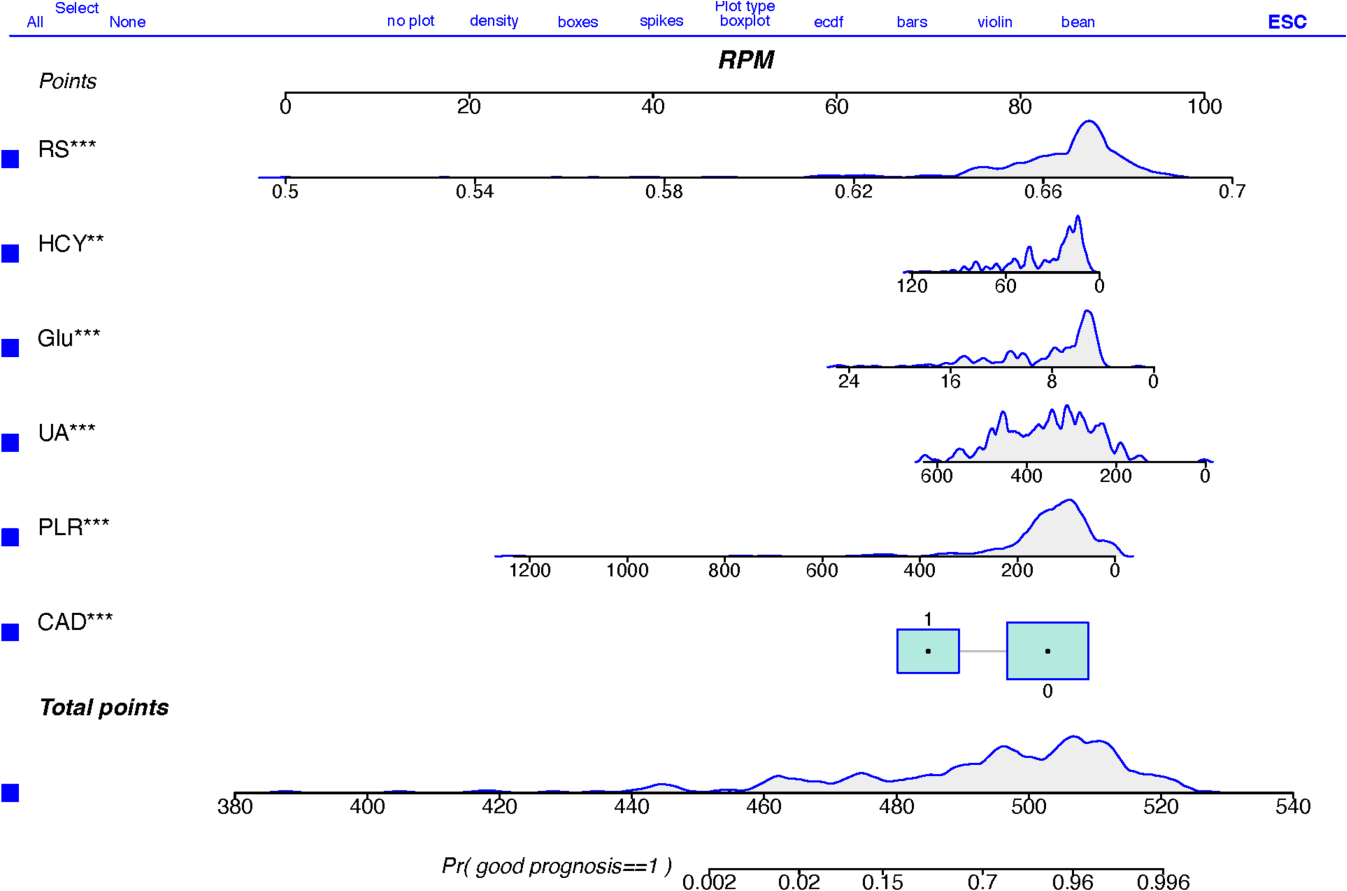
A nomogram based on clinical-radiomics models for predicting AIS outcomes.

Table 4 presents the results of the multivariate logistic regression analysis, identifying significant variables (*p* < 0.05) such as Coronary Artery Disease (CAD), White Blood Cell count (WBC), Platelet-to-Lymphocyte Ratio (PLR), Uric Acid (UA), Glucose (Glu), Homocysteine (HCY), and the Radiomics Score (RS). These variables have been pinpointed as independent predictors for clinical functional outcomes. Utilizing these determinants, we constructed a radiomics nomogram within the training set, creating a clinical-radiomics comprehensive prediction model (illustrated in Figure 4).

**Table 4:** Multivariate logistic regression.

Variable	OR(95%CI)	*p*-value	
CAD	0.108(0.043, 0.255)	<0.001	***
PLR	0.991 (0.985, 0.996)	<0.001	***
UA	0.992 (0.988, 0.995)	<0.001	***
Glu	0.789 (0.709, 0.874)	<0.001	***
HCY	0.971 (0.954, 0.989)	<0.001	***
RS	1.86e + 38 (9.48e + 24, 3.30e + 55)	<0.001	***

Significant codes: 0; “***”0.001; “**”0.01; “*”0.05;“.”0.1; “ ”1.

The nomogram is meticulously designed, highlighting the relative importance of variables identified through multivariate regression analysis. A scale is displayed at the top of the column chart, with predictive model variables listed on the left. Each independent variable is assigned a score, correlating to specific values, with the length of each segment indicating its contributory weight to the outcome event. Hence, longer segments underscore a variable’s heightened significance. By aggregating scores for individual variables, a total score is achievable. This cumulative score facilitates the determination of the linear predictor and predicted probability for Acute Ischemic Stroke (AIS) prognosis through the scale provided below. A prediction value of 1 suggests a favorable prognosis, whereas 0 implies a less favorable prognosis.

### 3.4 Performance of the combined clinical-radiomics model

**Figure 5:**
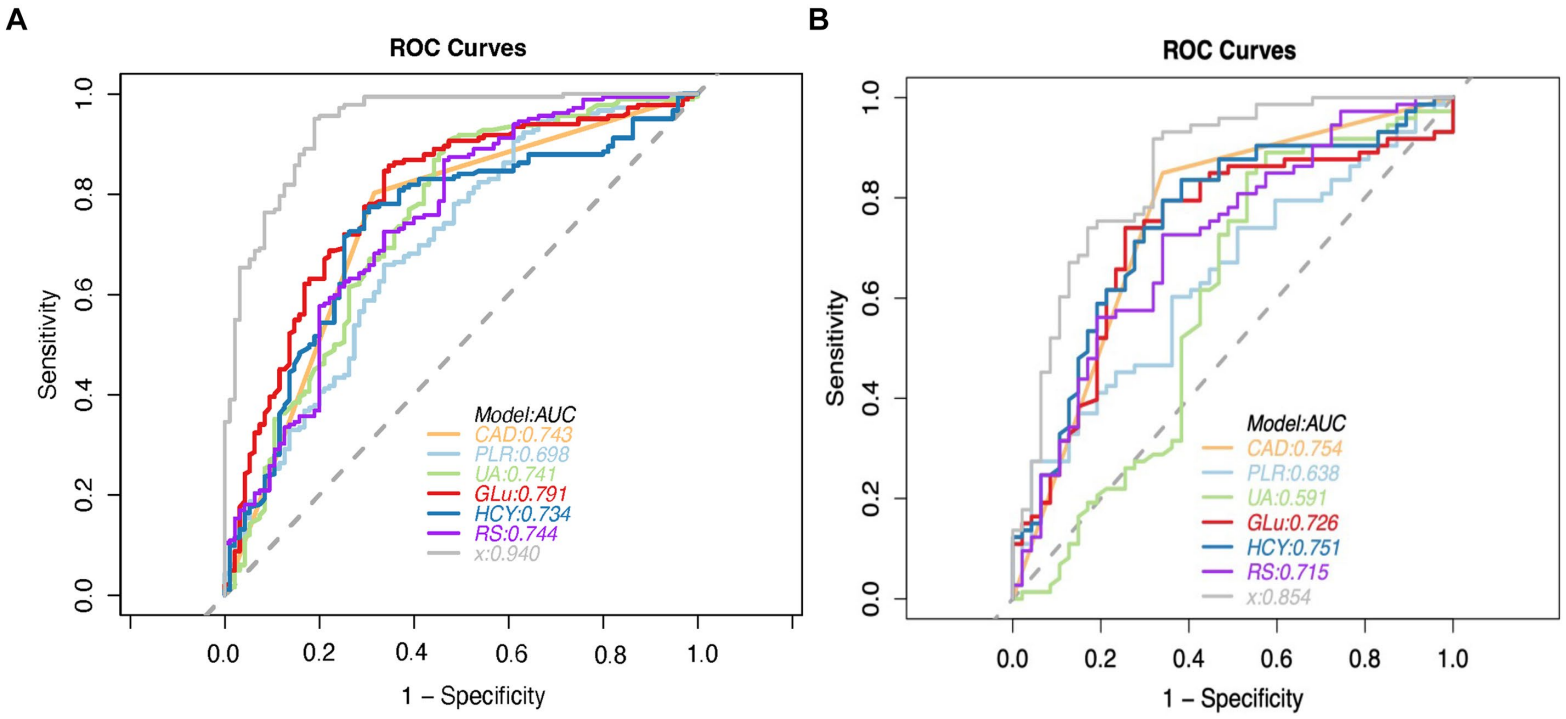
ROC curve analysis of predictive models for modified rankin scale (MRS) outcomes in the training **(A)** and validation **(B)** cohorts, with the clinical-radiomics model highlighted. CAD = coronary artery disease, PLR = platelet-lymphocyte ratio, UA = serum uric acid level, GLU = fasting plasma glucose, HCY = homocysteine, RS = Radiomics Signature, x = Clinical-Radiomics model.

The performance of the combined clinical-radiomics model was meticulously evaluated using Receiver Operating Characteristic (ROC) curves. This comprehensive model exhibited impressive predictive accuracy for Acute Ischemic Stroke (AIS) outcomes, as evidenced by Area Under the Curve (AUC) scores of 0.940 (95% Confidence Interval [CI]: 0.912–0.969) in the training cohort and 0.853 (95% CI: 0.781–0.926) in the validation cohort. Notably, in the validation cohort, the clinical-radiomics model achieved high sensitivity (91.8%) and moderate specificity (68.1%), indicating its robust ability to accurately predict patient outcomes. The Positive Predictive Value (PPV) of the clinical-radiomics approach in the validation set was notably high, at approximately 81.7%. The Radiomics Score (RS) and the clinical-radiomics models yielded AUC values of 0.715 (95% CI: 0.619–0.810) and 0.854 (95% CI: 0.781–0.926), respectively, as illustrated in Figure 5.

Clinical variables such as Coronary Artery Disease (CAD), Platelet-to-Lymphocyte Ratio (PLR), Uric Acid (UA), Glucose (Glu), and Homocysteine (HCY) demonstrated significant predictive utility, as detailed in Table 5. The DeLong test revealed no significant differences in the performance of these three individual models (all *p* > 0.05), underscoring the consistency of the predictive capability across the models.

**Table 5:** Predictive performance of three models in the training and validation cohorts.

Model	Training cohort (*n* = 277)					
	AUC (95% CI^*^)	Sensitivity	Specificity	Accuracy	PPV^*^	NPV^*^
CAD	0.743(0.688-0.798)	0.802	0.684	0.762	0.83	0.643
PLR	0.698(0.631–0.765)	0.659	0.663	0.66	0.789	0.504
UA	0.741(0.676–0.806)	0.907	0.526	0.776	0.786	0.746
Glu	0.791(0.734–0.849)	0.857	0.653	0.787	0.825	0.705
HCY	0.734(0.669–0.796)	0.774	0.694	0.747	0.829	0.617
Radiomics model	0.744(0.681–0.808)	0.868	0.536	0.755	0.782	0.68
Clinical-radiomics model	0.940(0.912–0.969)	0.951	0.811	0.903	0.906	0.895
**Model**	**Validation cohort (**n* *= 120)**					
	**AUC (95% CI)**	**Sensitivity**	**Specificity**	**Accuracy**	**PPV**	**NPV**
CAD	0.754(0.675–0.834)	0.85	0.66	0.775	0.795	0.738
PLR	0.591(0.538–0.738)	0.603	0.638	0.617	0.721	0.508
UA	0.726(0.477–0.705)	0.468	0.849	0.7	0.713	0.667
Glu	0.791(0.630–0.822)	0.74	0.745	0.742	0.818	0.648
HCY	0.751(0.659–0.842)	0.795	0.66	0.742	0.784	0.674
Radiomics model	0.714(0.619–0.810)	0.726	0.66	0.7	0.768	0.608
Clinical-radiomics model	0.854(0.781–0.926)	0.918	0.681	0.825	0.817	0.842

^*^CI, confidence interval; PPV, Positive predictive value; NPV, Negative predictive value.

### 3.5 Clinical usefulness and calibration curves for the clinical-radiomics

**Figure 6:**
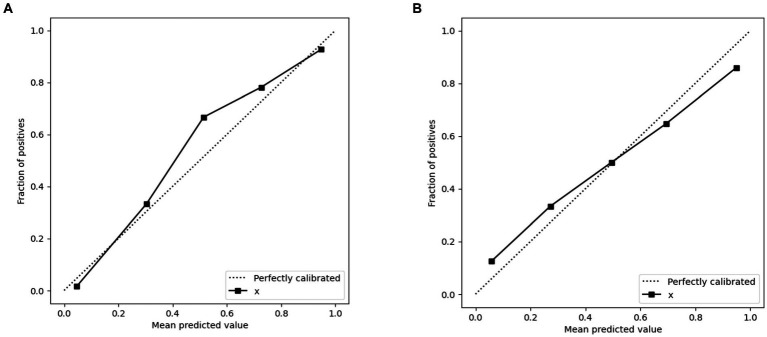
Calibration curves of the clinical-radiomics model in the training set **(A)** and validation set **(B)** for predicting AIS outcomes. x = Clinical-Radiomics model.

When contrasted with single-scale prediction models, the clinical-radiomics model showcased a superior capability in discriminating performance evaluation. As depicted in Figure 6, calibration plots demonstrated a commendable congruence between the model’s predictions and the actual clinical outcomes for Acute Ischemic Stroke (AIS). This alignment indicates that the clinical-radiomics model provides a significantly enhanced net benefit over traditional single-scale models, underscoring its greater clinical utility.

## 4 Discussion

Clinical predictive models are increasingly utilized in acute ischemic stroke management, serving as a critical role in prognosis and diagnostic support. Such as the widely recognized modified Rankin Scale (mRS), which could provide prognostic insights based on thorough patient evaluations, have gained broad acceptance in clinical settings. Historically, studies on ischemic stroke prognosis have predominantly utilized non-imaging data, employing various statistical models and machine learning algorithms such as linear regression, support vector machine (SVM), k-nearest neighbors (KNN), logistic regression, decision trees, k-means clustering, random forests (RF), naive Bayes, dimensionality reduction techniques, and gradient boosting. However, these methods have limitations, particularly their dependence on basic clinical data without incorporating detailed biomarkers or complex imaging findings. Such limitations may impede a comprehensive understanding of the multifaceted nature of the disease. Moreover, despite the importance of these thorough assessments, they may not adequately account for individual variability and specific lesion characteristics inherent in the nonlinear dynamics and subjective assessments of stroke prognosis evaluation systems, posing challenges in reflecting the dynamic progression of the condition (16, 17). The advancement of precision medicine calls for more sophisticated and precise methods capable of navigating the intricacies of ischemic stroke prognosis. In this context, artificial intelligence (AI) models emerge as potent tools, offering the potential for more tailored and accurate prognostic predictions.

The progression of precision medicine necessitates more sophisticated and precise methodologies capable of addressing the intricate nature of ischemic stroke prognosis. As a result, there is a growing focus on integrating diverse data sources, such as clinical observations, radiomics features, and biomarkers, to construct predictive models that provide improved accuracy, comprehensiveness, and personalization. Technological advancements have facilitated an increase in studies integrating imaging data (e.g., CT and MRI scans) with state-of-the-art artificial intelligence machine learning techniques to enhance the accuracy and sensitivity of stroke prognosis prediction models. This evolving paradigm highlights the comprehensive utilization of diverse data types, especially imaging data, creating new opportunities for advancing stroke prognosis research and achieving more detailed and precise assessments of patient prognoses in the medical domain (18).

Radiomics and artificial intelligence hold great promise in the management of acute stroke. Cranial CT is the standard screening tool post-stroke, with the Alberta Stroke Program Early CT score (ASPECTS) being a rapid, straightforward, and reliable method for assessing early ischemic changes in patients with ischemic stroke, which is crucial for predicting treatment outcomes and prognosis (19). Nicolae et al. (20) retrospectively analyzed 340 patients revealed that ASPECTS effectively predicts the prognosis of patients with acute ischemic stroke (AIS), with lower scores indicating a larger infarct volume, particularly in diabetic and elderly patients. Chen et al. (21) retrospectively analyzed the CT images of 276 AIS patients, confirming the reliability and accuracy of automated ASPECTS scoring software. Hulin Kuang et al. (22) introduced EIS-Net, a novel multi-task learning network capable of simultaneously segmenting early infarcts and scoring ASPECTS on non-contrast CT images, offering performance comparable to expert assessments in a rapid manner. Masaki et al. (23) developed a deep learning-based automated ASPECTS calculation software utilizing the 3D-BHCA algorithm, demonstrating higher accuracy and efficiency than traditional methods, which could assist physicians in formulating superior treatment plans. Additionally, Qi et al. (24) explored apparent diffusion coefficient (ADC) image signal changes and their quantitative assessments across 207 acute ischemic stroke patients. Their findings suggested these analyses could act as crucial references for estimating acute ischemic stroke volume, thus offering valuable diagnostic insights for clinical practice. Ma’s study (25) corroborated these observations. Future research may integrate multimodal imaging with clinical factors, offering more possibilities for precision diagnosis.

Cerebrovascular malformations, commonly arising from the abnormal development of intracranial vessels, can precipitate severe events such as stroke. Moyamoya disease (MMD), predominantly affecting children and adults, is marked by arterial stenosis and occlusion, with the potential to cause conditions like epilepsy and cognitive delays in pediatric patients. Despite advancements in modern imaging facilitating diagnosis, therapeutic options for MMD are limited, underscoring the critical importance of early detection and intervention to improve patient outcomes (26). The emergence of artificial intelligence (AI), particularly in the realms of deep learning and machine learning, has introduced novel approaches to MMD diagnosis.

In Kim’s study (27), they utilized deep learning and convolutional neural network (CNN) techniques to analyze cranial images from 345 diagnosed MMD patients and 408 control subjects for the detection of MMD. CNN for the analysis of images from MMD patients and controls demonstrated impressive levels of accuracy, sensitivity, and specificity, underscoring the potential of AI in medical imaging.

Qin et al. (28) utilized machine learning models to analyze DSA images and predict mean transit time in MMD or Moyamoya syndrome (MMS) patients, achieving high accuracy in specific brain regions, which could offer significant insights for clinical diagnosis and treatment. Collectively, these studies suggest a promising role for AI in refining MMD diagnosis and treatment strategies.

Multimodal MRI, incorporating a variety of imaging sequences such as DWI, FLAIR, susceptibility-weighted imaging (SWI), and T1w and T2w, offers a rich source of biological insights. This comprehensive imaging approach enables physicians to thoroughly understand the characteristics and extent of lesions, thereby enhancing the precision of disease diagnoses. Additionally, employing radiomics and machine learning techniques, multimodal MRI facilitates the automated extraction and analysis of extensive imaging features. By amalgamating data from different MRI sequences, this strategy supports the development of predictive models capable of accurately determining patient outcomes. The confluence of multiple imaging modalities with sophisticated analytical methods promises more accurate and personalized medical evaluations.

In another significant effort, Quan et al. (14) derived radiomics features from fluid-attenuated inversion recovery (FLAIR) and ADC images across 753 cases, demonstrating their utility in forecasting clinical outcomes in acute ischemic stroke (AIS) patients. External validation showed promising area-under-the-curve (AUC) values across various models, with the combined ADC and FLAIR radiomics model significantly enhancing predictive accuracy for adverse outcomes.

A meta-analysis by Hanna Maria Dragoş et al. (29), reviewing 150 articles, suggests models that merge clinical and imaging features more effectively predict disability outcomes in stroke patients at 3 and 6 months post-event. Wang’s study (30) established three radiomics models and a comprehensive nomogram that integrated clinical features and radiomic signatures, showing robust predictive performance for post-thrombolytic ischemic stroke prognosis.

To predict ischemic stroke outcomes, Yu et al. (31) utilized a machine learning model integrating multimodal images, including DWI, ADC, FLAIR, SWI, and T1w. This model achieved notable performance metrics, with an accuracy of 0.831, sensitivity of 0.739, specificity of 0.902, an F1 score of 0.788, and an AUC of 0.902. Radiomic features extracted using the LightGBM model from multimodal MRI effectively forecast stroke prognosis, demonstrating the model’s high predictive value for clinical outcomes in acute stroke patients. This underscores the potential of multimodal imaging in making precise prognosis predictions for ischemic stroke.

While the feasibility of using machine learning based on radiomics alongside clinical factors for predicting acute ischemic stroke outcomes is recognized, it is crucial to be mindful of possible biases in participant selection across studies. Zhou et al. (32) predicted acute ischemic stroke outcomes by blending radiomic features from multimodal imaging with clinical factors. Their clinical-radiomic nomogram showed superior ROC AUCs in both training and validation groups, achieving 0.868 and 0.890, respectively, surpassing models based solely on clinical or radiomic data. Despite its utility, this study’s single-center nature and restriction to DWI and ADC sequences without considering other modalities may introduce biases.

Future research should aim for broader and more diverse datasets, implement stringent study methodologies, and address potential biases to solidify the reliability and applicability of predictive models. Including data from multiple centers and varied patient demographics will strengthen the external validity of research outcomes, paving the way for advancements in personalized stroke management.

Diverging from prior research, our study utilized extensive datasets and integrated radiomic features from multimodal MRI (T1w, T2w, DWI) with clinical risk factors to develop a clinical-radiomic model to forecast the prognosis of acute ischemic stroke. This model exhibited enhanced performance in the validation set, outperforming individual imaging features or clinical factors in discriminative capacity, calibration, and clinical applicability. The model’s performance metrics in the training set were noteworthy: sensitivity reached 0.951, specificity 0.811, and accuracy 0.903. Furthermore, in the validation set, sensitivity was 0.918, specificity 0.681, and accuracy 0.825. Despite some variability in these metrics, our model consistently offers valuable insights for clinicians in making informed decisions regarding the treatment and prognosis of acute ischemic stroke.

This research leveraged a broad spectrum of imaging modalities for feature extraction, including T1w, T2w, and both raw and processed DWI images. Twenty-one radiomic features were extracted, covering dimensions such as shape, energy, texture, and various gray-level matrices (GLCM, GLRLM, GLSZM, GLDM). These features provide a comprehensive portrayal of ischemic stroke heterogeneity, offering a sophisticated understanding of stroke pathology. Notably, elevated values in these feature analyses correlate with poorer patient outcomes, enabling precise and quantifiable assessments of imaging characteristics linked to ischemic stroke prognosis.

It’s worth noting that our study revealed multivariate logistic regression analysis revealed coronary heart disease, uric acid levels, blood glucose levels, homocysteine, and the platelet-to-lymphocyte ratio (PLR) as independent risk factors affecting stroke prognosis, which align with clinical realities.

Extensive research supports that high blood glucose levels are an independent risk factor for stroke, increasing susceptibility to ischemic stroke by 2–4 times (33). The strong correlation between type 2 diabetes and stroke risk is well-documented across various studies (33, 34). Effective blood glucose management is crucial for reducing the risk of diabetes and its complications, notably the increased risk of stroke.

Coronary heart disease and ischemic stroke often overlap in their underlying mechanisms, with individuals with coronary heart disease at higher risk of cardiovascular events after a stroke. This interaction can lead to complex multi-organ impairment and hinder optimal recovery. A comprehensive treatment approach is essential to manage these interconnected conditions and improve patient outcomes (35).

Uric acid, a product of purine breakdown, has dual roles: it scavenges free radicals and promotes neuronal glutathione synthesis, offering neuroprotection. Elevated uric acid levels are linked to stroke risk due to its oxidative properties, though it also has neuroprotective effects. Maintaining the balance of uric acid is crucial to managing stroke risk (36).

Elevated homocysteine levels, often associated with deficiencies in folate, vitamin B6, and B12, significantly increase stroke risk. For every 5 umol/L rise in homocysteine levels, the risk of stroke escalates by 95%. Higher levels are also linked to early neurological deterioration and increased risk of stroke recurrence and mortality. Monitoring homocysteine levels is crucial in mitigating stroke risk (37).

The platelet-to-lymphocyte ratio (PLR), derived from platelet and lymphocyte counts, serves as an inflammation marker and prognostic indicator for disease progression (38). PLR has predictive value in various conditions, including cancer. In acute ischemic stroke (AIS), heightened PLR levels correlate with larger infarcts and poorer prognosis. PLR also predicts clinical outcomes at 90 days, making it a valuable prognostic biomarker in AIS scenarios (39).

## 5 Limitation

While this research offers promising insights, it is imperative to recognize its limitations. The study’s retrospective design introduces the potential for selection bias and the influence of confounding variables. Furthermore, the study is constrained by a relatively small participant pool and a limited scope for external validation. Future research would benefit from prospective, multicenter studies encompassing larger cohorts and broader validation efforts to bolster the findings’ robustness. Additionally, this investigation’s treatment of ischemic stroke etiology, especially concerning cerebral infarction locations, lacks granularity. The reliance on manual delineation for regions of interest (ROI) could introduce subjectivity; thus, subsequent studies might enhance accuracy by adopting advanced software solutions, refining training protocols, and minimizing subjective bias. The inconsistency in standard head MRI sequences across various institutions and divergent institutional protocols posed challenges in evaluating a comprehensive range of MRI sequences. Future endeavors should focus on improving patient engagement, augmenting the collection of multimodal MRI data (e.g., FLAIR, ADC, SWAN), and constructing sophisticated models to overcome these limitations. Importantly, the initial study cohort was composed exclusively of patients who did not receive ischemic reperfusion therapy. Comparative analyses of patients undergoing ischemic reperfusion therapy vs. those who do not could provide valuable insights into the predictive utility of radiological markers and clinical indicators in treated acute ischemic stroke patients.

## 6 Conclusion

In conclusion, this study demonstrates that the incorporation of radiomic features from T1-weighted (T1w), T2-weighted (T2w), and diffusion-weighted imaging (DWI) with clinical parameters into a cohesive clinical-radiomics model significantly improves the predictive accuracy for the prognosis and treatment responses in ischemic stroke. This advancement fosters a deeper comprehension of therapeutic impacts, prognostic evaluations, and clinical assessments, offering invaluable insights for medical professionals in diagnosis, therapeutic intervention, and rehabilitation. The synergy between radiomic attributes and clinical information heralds a promising avenue for enhancing personalized medicine strategies and elevating patient care outcomes in ischemic stroke.

## Data availability statement

The raw data supporting the conclusions of this article will be made available by the authors, without undue reservation.

## Ethics statement

The studies involving human participants were reviewed and approved by the Institutional Review Board at Xi’an Central Hospital and Tongchuan Mining Bureau Hospital. Written informed consent was not required due to the retrospective nature of the study.

## Author contributions

KG: Writing – original draft, Writing – review & editing, Conceptualization, Data curation, Formal analysis, Project administration, Resources, Software, Validation, Visualization. BZ: Data curation, Writing – review & editing. RL: Data curation, Investigation, Writing – review & editing. JX: Data curation, Investigation, Writing – review & editing. QW: Methodology, Software, Visualization, Writing – review & editing. KC: Data curation, Investigation, Writing – review & editing. YS: Data curation, Investigation, Writing – review & editing. JL: Investigation, Writing – review & editing, Project administration, Supervision. WC: Supervision, Writing – review & editing, Project administration. ZL: Project administration, Supervision, Writing – review & editing. ZD: Writing – review & editing, Funding acquisition. NG: Writing – original draft, Writing – review & editing, Funding acquisition.

## References

[1] GBD 2019 Diseases and Injuries Collaborators. Global burden of 369 diseases and injuries in 204 countries and territories, 1990-2019: a systematic analysis for the global burden of disease study 2019. Lancet. (2020) 396:1204–22. doi: 10.1016/S0140-6736(20)30925-9 33069326 PMC7567026

[2] National Institute of Neurological Disorders and Stroke rt-PA Stroke Study Group. Tissue plasminogen activator for acute ischemic stroke. N Engl J Med. 333:1581–7. doi: 10.1056/NEJM199512143332401 7477192

[3] Hacke W Kaste M Bluhmki E Brozman M Dávalos A Guidetti D. Thrombolysis with Alteplase 3 to 4.5 hours after acute ischemic stroke. N Engl J Med Overseas Ed. 359:1317–29. doi: 10.1056/NEJMoa0804656 18815396

[4] Bracard S Ducrocq X Mas JL Soudant M Oppenheim C Moulin T. Investigators T: mechanical thrombectomy after intravenous alteplase versus alteplase alone after stroke (THRACE): a randomised controlled trial. Lancet Neurol. (2016) 15:1138–47. doi: 10.1016/S1474-4422(16)30177-6, PMID: 27567239

[5] Yao M Ren Y Jia Y Xu J Wang Y Zou K. Projected burden of stroke in China through 2050. Chin Med J. (2023) 136:1598–605. doi: 10.1097/CM9.0000000000002060, PMID: 36580638 PMC10325738

[6] Wang YJ Li ZX Gu HQ Zhai Y Jiang Y Zhao XQ. China stroke statistics 2019: a report from the National Center for healthcare quality Management in Neurological Diseases, China National Clinical Research Center for neurological Diseases, the Chinese Stroke Association, National Center for chronic and non-communicable disease control and prevention, Chinese Center for Disease Control and Prevention and Institute for global neuroscience and stroke collaborations. Stroke Vasc Neurol. (2020) 5:211–39. doi: 10.1136/svn-2020-000457, PMID: 32826385 PMC7548521

[7] Eslami V Tahsili-Fahadan P Rivera-Lara L Gandhi D Ali H Parry-Jones A. Influence of intracerebral hemorrhage location on outcomes in patients with severe intraventricular hemorrhage. Stroke. (2019) 50:1688–95. doi: 10.1161/STROKEAHA.118.024187, PMID: 31177984 PMC6771028

[8] Johnston KC Wagner DP Haley EC Jr Connors AF Jr. Stroke RIRToTMiA: combined clinical and imaging information as an early stroke outcome measure. Stroke. (2002) 33:466–72. doi: 10.1161/hs0202.102881, PMID: 11823654 PMC2749233

[9] Zhao J Feng J Ma Q Li C Qiu F. Prognostic value of inflammation biomarkers for 30-day mortality in critically ill patients with stroke. Front Neurol. (2023) 14:1110347. doi: 10.3389/fneur.2023.1110347, PMID: 36814998 PMC9939760

[10] van Timmeren JE Cester D Tanadini-Lang S Alkadhi H Baessler B. Radiomics in medical imaging-"how-to" guide and critical reflection. Insights Imaging. (2020) 11:91. doi: 10.1186/s13244-020-00887-2, PMID: 32785796 PMC7423816

[11] Rogers W Thulasi Seetha S Refaee TAG Lieverse RIY Granzier RWY Ibrahim A. Radiomics: from qualitative to quantitative imaging. Br J Radiol. (2020) 93:20190948. doi: 10.1259/bjr.20190948, PMID: 32101448 PMC7362913

[12] Yang Y Tang L Deng Y Li X Luo A Zhang Z. The predictive performance of artificial intelligence on the outcome of stroke: a systematic review and meta-analysis. Front Neurosci. (2023) 17:1256592. doi: 10.3389/fnins.2023.1256592, PMID: 37746141 PMC10512718

[13] Chen Q Xia T Zhang M Xia N Liu J Yang Y. Radiomics in stroke neuroimaging: techniques, applications, and challenges. Aging Dis. (2021) 12:143–54. doi: 10.14336/AD.2020.0421, PMID: 33532134 PMC7801280

[14] Quan G Ban R Ren JL Liu Y Wang W Dai S. FLAIR and ADC image-based Radiomics features as predictive biomarkers of unfavorable outcome in patients with acute ischemic stroke. Front Neurosci. (2021) 15:730879. doi: 10.3389/fnins.2021.730879, PMID: 34602971 PMC8483716

[15] Zhang Y Zhuang Y Ge Y Wu PY Zhao J Wang H. MRI whole-lesion texture analysis on ADC maps for the prognostic assessment of ischemic stroke. BMC Med Imaging. (2022) 22:115. doi: 10.1186/s12880-022-00845-y, PMID: 35778678 PMC9250246

[16] Wang L Liu Z Liang R Wang W Zhu R Li J. Comprehensive. Machine-learning survival framework develops a consensus model in large-scale multicenter cohorts for pancreatic cancer. eLife. (2022) 11:150. doi: 10.7554/eLife.80150 PMC9596158 36282174

[17] Alexandra Z Dongyu L Rhema V Kalyan V: Sibyl: understanding and addressing the. Usability challenges of machine learning in high-stakes decision making. IEEE Transactions on Visualization and Computer Graphics. arXiv [Preprint]. arXiv:2103.02071v2. (2021). 10.1109/TVCG.2021.3114864 34587081

[18] Guo Y Yang Y Cao F Li W Wang M Luo Y. Novel. Survival features generated by clinical text information and Radiomics features may improve the prediction of ischemic stroke outcome. Diagnostics. (2022) 12:12(7). doi: 10.3390/diagnostics12071664 PMC9324145 35885568

[19] Barber PA Demchuk AM Zhang J Buchan AM. Validity and reliability of a quantitative computed tomography score in predicting outcome of hyperacute stroke before thrombolytic therapy. ASPECTS study group. Alberta stroke Programme early CT score. Lancet. (2000) 355:1670–4. doi: 10.1016/S0140-6736(00)02237-6, PMID: 10905241

[20] Pop NO Tit DM Diaconu CC Munteanu MA Babes EE Stoicescu M. The Alberta stroke program early CT score (ASPECTS): a predictor of mortality in acute ischemic stroke. Exp Ther Med. (2021) 22:1371. doi: 10.3892/etm.2021.10805, PMID: 34659517 PMC8515558

[21] Chen Z Shi Z Lu F Li L Li M Wang S. Validation of two automated ASPECTS software on non-contrast computed tomography scans of patients with acute ischemic stroke. Front Neurol. (2023) 14:1170955. doi: 10.3389/fneur.2023.1170955, PMID: 37090971 PMC10116051

[22] Kuang H Menon BK Sohn SI Qiu W. EIS-net: segmenting early infarct and scoring ASPECTS simultaneously on non-contrast CT of patients with acute ischemic stroke. Med Image Anal. (2021) 70:101984. doi: 10.1016/j.media.2021.101984, PMID: 33676101

[23] Naganuma M Tachibana A Fuchigami T Akahori S Okumura S Yi K. Alberta stroke program early CT score calculation using the deep learning-based brain hemisphere comparison algorithm. J Stroke Cerebrovasc Dis. (2021) 30:105791. doi: 10.1016/j.jstrokecerebrovasdis.2021.105791, PMID: 33878549

[24] Qian Q Huang HT Xu L Jin P Lin M. Prediction of infarct lesion volumes by processing magnetic resonance apparent diffusion coefficient maps in patients with acute ischemic stroke. J Stroke Cerebrovasc Dis. (2016) 25:2821–7. doi: 10.1016/j.jstrokecerebrovasdis.2016.07.041, PMID: 27618198

[25] Ma L Gao PY Hu QM Lin Y Jing LN Xue J. Effect of baseline magnetic resonance imaging (MRI) apparent diffusion coefficient lesion volume on functional outcome in ischemic stroke. Neurol Res. (2011) 33:494–502. doi: 10.1179/016164111X13007856084124, PMID: 21669118

[26] Nicolae O Pop DCZ Pantiș C Mekeres F. Clinicopathological evaluation of Moyamoya disease. Case report and review of literature. Roman J Milit Med. (2020) CXXIII:5. doi: 10.55453/rjmm.2020.123.2.5

[27] Kim T Heo J Jang DK Sunwoo L Kim J Lee KJ. Machine learning for detecting moyamoya disease in plain skull radiography using a convolutional neural network. EBioMedicine. (2019) 40:636–42. doi: 10.1016/j.ebiom.2018.12.043, PMID: 30598372 PMC6413674

[28] Qin K Guo Z Peng C Gan W Zhou D Chen G. Prediction of the mean transit time using machine learning models based on radiomics features from digital subtraction angiography in moyamoya disease or moyamoya syndrome-a development and validation model study. Cardiovasc Diagn Ther. (2023) 13:879–92. doi: 10.21037/cdt-23-151, PMID: 37941836 PMC10628422

[29] Dragos HM Stan A Pintican R Feier D Lebovici A Panaitescu PS. MRI Radiomics and predictive models in assessing ischemic stroke outcome-a systematic review. Diagnostics. (2023) 13:857. doi: 10.3390/diagnostics13050857 36900001 PMC10000411

[30] Wang H Sun Y Ge Y Wu PY Lin J Zhao J. A clinical-Radiomics nomogram for functional outcome predictions in ischemic stroke. Neurol Ther. (2021) 10:819–32. doi: 10.1007/s40120-021-00263-2, PMID: 34170502 PMC8571444

[31] Yu H Wang Z Sun Y Bo W Duan K Song C. Prognosis of ischemic stroke predicted by machine learning based on multi-modal MRI radiomics. Front Psych. (2022) 13:1105496. doi: 10.3389/fpsyt.2022.1105496 PMC9868394 36699499

[32] Zhou Y Wu D Yan S Xie Y Zhang S Lv W. Feasibility of a clinical-Radiomics model to predict the outcomes of acute ischemic stroke. Korean J Radiol. (2022) 23:811–20. doi: 10.3348/kjr.2022.0160, PMID: 35695316 PMC9340229

[33] The Emerging Risk Factors Collaboration. Diabetes mellitus, fasting blood glucose concentration, and risk of vascular disease: a collaborative meta-analysis of 102 prospective studies. Lancet. (2010) 375:2215–22. doi: 10.1016/S0140-6736(10)60484-9, PMID: 20609967 PMC2904878

[34] Guo L Yu M Zhong J Wu H Pan J Gong W. Stroke risk among patients with type 2 diabetes mellitus in Zhejiang: a population-based prospective study in China. Int J Endocrinol. (2016) 2016:1–8. doi: 10.1155/2016/6380620 PMC4923572 27403161

[35] Yousufuddin M Bartley AC Alsawas M Sheely HL Shultz J Takahashi PY. Impact of multiple chronic conditions in patients hospitalized with stroke and transient ischemic attack. J Stroke Cerebrovasc Dis. (2017) 26:1239–48. doi: 10.1016/j.jstrokecerebrovasdis.2017.01.015 28285088

[36] Qiao T Wu H Peng W. The relationship between elevated serum uric acid and risk of stroke in adult: an updated and dose-response Meta-analysis. Front Neurol. (2021) 12:674398. doi: 10.3389/fneur.2021.674398, PMID: 34526951 PMC8435906

[37] The Homocyteine Studies Collaboation. Homocysteine and risk of ischemic heart Diease and stroke a meta-analysis. JAMA. (2015) 288:2015. doi: 10.1001/jama.288.16.2015 12387654

[38] Altintas O Altintas MO Tasal A Kucukdagli OT Asil T. The relationship of platelet-to-lymphocyte ratio with clinical outcome and final infarct core in acute ischemic stroke patients who have undergone endovascular therapy. Neurol Res. (2016) 38:759–65. doi: 10.1080/01616412.2016.1215030, PMID: 27477691

[39] Chen C Gu L Chen L Hu W Feng X Qiu F. Neutrophil-to-lymphocyte ratio and platelet-to-lymphocyte ratio as potential predictors of prognosis in acute ischemic stroke. Front Neurol. (2021) 11:525621. doi: 10.3389/fneur.2020.525621 33569032 PMC7868420

